# Insights into Chemoreceptor MCP2201-Sensing D-Malate

**DOI:** 10.3390/ijms26104902

**Published:** 2025-05-20

**Authors:** Rui Cui, Jie Li, Yuan Hong, Lu Guo, Yun-Hao Wang, Yi-Fei Bai, De-Feng Li

**Affiliations:** 1 State Key Laboratory of Microbial Diversity and Innovative Utilization, Institute of Microbiology, Chinese Academy of Sciences, Beijing 100101, China; cuir@im.ac.cn (R.C.); lijie244@mails.ucas.ac.cn (J.L.); guolu@im.ac.cn (L.G.); yb2365@nyu.edu (Y.-F.B.); 2 School of Life Sciences, University of Chinese Academy of Sciences, Beijing 100049, China; 3 National Laboratory of Biomacromolecules, CAS Center for Excellence in Biomacromolecules, Institute of Biophysics, Chinese Academy of Sciences, Beijing 100101, China; hongyuan11@mails.ucas.ac.cn; 4 Key Laboratory of Pesticide & Chemical Biology of Ministry of Education, School of Life Sciences, Central China Normal University, Wuhan 430079, China; yunhao@ccnu.edu.cn

**Keywords:** chemoreceptor, chemoattractant, chemorepellent, enantiomer

## Abstract

Bacterial chemoreceptors sense extracellular stimuli and drive bacteria toward a beneficial environment or away from harm. Their ligand-binding domains (LBDs) are highly diverse in terms of sequence and structure, and their ligands cover various chemical molecules that could serve as nitrogen, carbon, and energy sources. The mechanism of how this diverse range of LBDs senses different ligands is essential to signal transduction. Previously, we reported that the chemoreceptor MCP2201 from *Comamonas testosteroni* CNB-1 sensed citrate and L-malate, altered the ligand-free monomer–dimer equilibrium of LBD to citrate-bound monomer (with limited monomer) and L-malate-bound dimer, and triggered positive and negative chemotactic responses. Here, we present our findings, showing that D-malate binds to MCP2201, induces LBD dimerization, and triggers the chemorepellent response exactly as L-malate did. A single site mutation, T105A, can alter the D-malate-bound LBD dimer into a monomer–dimer equilibrium and switch the negative chemotactic response to D-malate to a positive one. Differences in attractant-bound LBD oligomerization, such as citrate-bound wildtype LBD monomer and D-malate-bound T105A dimer, indicated that LBD oligomerization is a consequence of signal transduction instead of a trigger. Our study expands our knowledge of chemoreceptor-sensing ligands and provides insight into the evolution of bacterial chemoreceptors.

## 1. Introduction

Via chemotaxis, bacteria sense extracellular stimuli, respond to the changing environment appropriately, and move toward a beneficial environment or away from harm. The chemotaxis signaling pathway is a non-canonical two-component signaling system, including methyl-accepting chemotaxis receptor proteins (MCPs), histidine kinase CheA, coupling protein CheW, and response regulatory protein CheY [1,2]. The canonical two-component signaling system typically consists of a sensor histidine kinase (HK) and a cognate response regulator (RR), where HK senses the signal, activates itself, and phosphorylates RR to activate the downstream signal [3,4]. In contrast, chemoreceptors, CheA, and CheY sense the chemical stimulus, phosphorylate CheY, and regulate the rotation of flagella, respectively [5,6]. Typical chemoreceptor proteins, such as the well-studied *E. coli* chemoreceptors Tar and Tsr, consist of three primary domains: a periplasmic ligand-binding domain (LBD), transmembrane helices (TMs), and a cytoplasmic signaling domain (SD) [5,6]. They form trimers-of-dimers and then assemble a chemotaxis array together with CheA and CheW [2,7,8]. They bind the chemical molecules at the ligand-binding domains, transduce the conformational change induced by the ligand binding into the cytoplasm via transmembrane helices, and regulate the activity of the histidine kinase CheA [9]. The response regulatory protein CheY is phosphorylated by activated CheA and then regulates bacterial movement by interacting with the flagellar motor [6,10].

Bacterial chemoreceptors share similar cytoplasmic signaling domains, which is considered a prerequisite for similar motility regulation in different bacteria. In contrast, their ligand-binding domains are highly diverse in sequences, structures, ligands, and signal generation. More than 80 types of LBDs have been found in bacterial genomes [11], including 4HB (four-helix bundle) [12,13], sCache (single-Calcium channels and chemotaxis receptors) [14,15], dCache (double Cache) [15,16,17], PAS (found in Per-Arnt-Sim proteins) [18,19], HBM (helical bundle domain) [20], CZB (chemoreceptor zinc binding) [21], GAF (found in cGMP-specific phosphodiesterases, adenylyl cyclases, and FhlA) [22,23], NIT (nitrate and nitrite sensing) [24], and PilJ (N-terminal domain of the type IV pilus chemoreceptor) [25,26,27]. Their ligands cover varieties of chemical molecules, which can serve as nitrogen sources, carbon sources, and energy sources and include amino acids [28], carbohydrates [29,30], di- and tricarboxylic acids [31], phosphate [32], urea [33,34], amine [35], O_2_ [36,37,38], and so on [39]. The extensively studied Tar from *E. coli* exclusively responds to chemoattractant aspartate via the 4HB LBD [40]. Aspartate binds to the LBD dimeric interface of Tar and alters the LBD monomer–dimer equilibrium into a dimeric organization by interacting with both monomers. Some bacterial chemoreceptors bind a class of molecules with similar structures and only trigger positive or negative chemotaxis. PctA (PA4309) from *P. aeruginosa* recognizes many chemoattractant amino acids via the dCache LBD [41,42,43]. Its LBD occurs as a monomer in ligand-bound and ligand-free forms and recognizes those amino acids mainly via their main chain atoms, providing a chance to accommodate a variety of side chains. TlpB from *H. pylori* binds urea and similar molecules, including hydroxyurea, formamide, and acetamide, via its sCache LBD dimer and then mediates pH sensing [35]. Further, some chemoreceptors recognize diverse ligands and trigger distinct chemotactic behaviors. CcmL from *Campylobacter jejuni* recognizes the chemoattractants isoleucine, malate, fumarate, and purine and the chemorepellents lysine, arginine, succinate, glucosamine, and thiamine via the dCache LBD [31]. The structure of the CcmL LBD complex with isoleucine reveals that CcmL recognizes the main chain amino group and carboxyl group of isoleucine and the side chain via hydrogen bonds and hydrophobic interactions, respectively. The mechanism for CcmL recognizing other ligands remains unclear.

MCP2201 from *Comamonas testosteroni* CNB-1 binds different di- and tricarboxylates, which are represented by TCA intermediates [44]. Citrate and L-malate have been found to be the chemoattractant and chemorepellent of MCP2201, respectively [44,45,46]. They were proposed to cause different orientations of signaling helix α4 after binding to the MCP2201 LBD and then trigger different chemotactic responses. The structure of MCP2201 LBD with citrate indicates that MCP2201 specifically only recognizes the D-malate moiety of citrate but not the additional acetic acid group, illustrating that MCP2201 only recognizes two carboxyl groups of either ligand. This suggests the reasoning for MCP2201 binding both di- and tricarboxylates. This observation suggests that MCP2201 could bind both L-malate and its enantiomer, a class of molecules ignored in our previous studies.

To reveal whether the chemoreceptors have the potential to recognize different enantiomers, we herein present the results of our study of the response of cells harboring MCP2201 to D-malate and the interaction between MCP2201 LBD and D-malate. We find that MCP2201 recognizes both malate enantiomers in similar but subtly different manners. A single residue mutation (T105A) could switch the negative chemotactic response to D-malate to a positive one. Our study expands the knowledge of the chemoreceptors’ ligand range and the evolution of bacterial chemoreceptors.

## 2. Results

### 2.1. D-Malate Is Recognized as a Chemorepellent by the MCP2201 LBD

**Figure 1:**
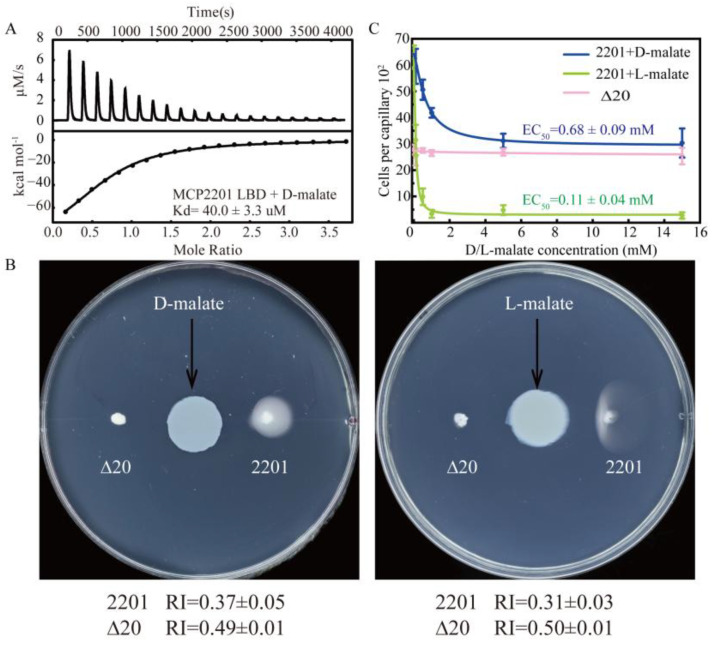
MCP2201 senses D-malate and triggers a chemorepellent response. (**A**) D-malate affinity measured by an isothermal titration calorimeter. (**B**) The chemotactic responses of CNB-1Δ20 cells harboring MCP2201, or not, toward D-malate (left) and L-malate (right) on the gradient soft-agar plate. (**C**) Chemotactic responses of MCP2201 toward different concentrations of D-malate (blue) and L-malate (green); Δ20 (pink) responds to different concentrations of D-malate in the capillary assays. Data were fitted to a logistic function. The experiments were repeated three times. The representative example is shown in (**A**,**B**).

In previous studies [45,46], we identified that MCP2201 senses L-malate and citrate as a chemorepellent and a chemoattractant, respectively. The ligand-binding domain of MCP2201 (MCP2201 LBD) recognized citrate predominantly by interacting with a D-malate moiety, suggesting the possibility that MCP2201 senses both malate enantiomers. MCP2201 LBD bound to D-malate with a dissociation constant (Kd) of 40.0 ± 3.3 μM (Figure 1A) and a binding number of 1.37 ± 0.08 in an isothermal titration calorimetry (ITC) assay. The affinity of D-malate was similar to those of L-malate (Kd of 18.8 ± 7.4 μM) and citrate (Kd of 95.8 ± 15.2 μM) [44,45,46].

We then checked whether CNB-1∆20/MCP2201 cells responded to D-malate using the gradient plate assay and capillary assays. In the gradient plate assay, the CNB-1∆20/MCP2201 and ∆20 cells responded to D-malate with chemotactic response index (RI) values of 0.37 ± 0.05 and 0.49 ± 0.01, respectively (Figure 1B). In this assay, RI values less than 0.48 and greater than 0.52 indicate repellent and attractant responses, respectively (see Methods for details). Therefore, the RI values suggested that D-malate triggered a repellent response in the same manner as L-malate. In the capillary assay, the number of CNB-1∆20/MCP2201 cells in the capillary decreased as D-malate concentrations increased (Figure 1C). In contrast, the number of cells in the ∆20 receptor-deficient strain did not decrease, accompanied by increasing D-malate concentrations. The observation confirmed that D-malate triggered a repellent response, with an EC_50_ value of 0.68 ± 0.09 mM. These findings collectively confirm that D-malate binds to the MCP2201 LBD and serves as a chemorepellent for MCP2201.

### 2.2. Both Malate Enantiomers Bind to the Same Pocket and Cause the Dimerization of LBD

**Figure 2:**
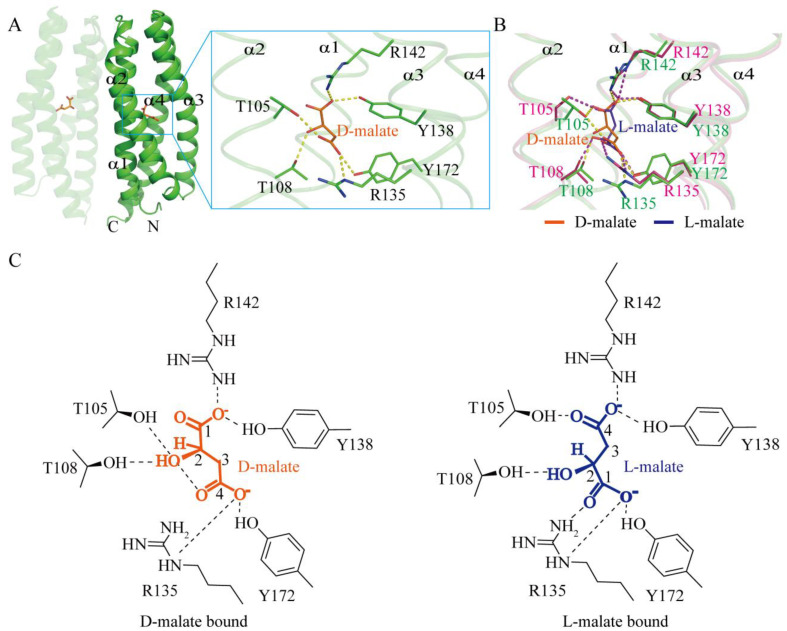
D-malate binds to the same pocket as L-malate, yet is subtly different. (**A**) D-malate-bound LBD dimer and the ligand binding pocket. (**B**) Superposition of D-malate (orange and green) and L-malate-binding pockets (blue and pink, PDB accession code 7WRM). Hydrogen bonds in the D-malate- and L-malate-bound structures are shown in yellow and purple, respectively, with binding motifs highlighted in green and pink. (**C**) Diagram comparison of D-malate (left) and L-malate (right) binding motifs.

To further elucidate the mechanism underlying D-malate’s role as a chemotactic repellent, we determined the crystal structure of D-malate-bound MCP2201 LBD at a resolution of 1.5 Å. Consistent with previous reports, the structure of D-malate-bound MCP2201 LBD shared the same four-helix bundle (α1: Q59-K87, α2: A91-L118, α3: P122-A150, and α4: A154-E193) with the L-malate-bound structure, with root-mean-square deviations (r.m.s.d) of 0.43 Å, and D-malate occupied the same binding position as L-malate and citrate. The pocket was formed by all four helices. More precisely, residues Y138 and R142 interacted with the 1′-carboxyl group, residue T108 interacted with the 2′-hydroxyl group, and residues R135 and Y172 interacted with the 4′-carboxyl group (Figure 2A,C). In contrast, those residue groups interacted with the 4′-carboxyl, 2′-hydroxyl, and 1′-carboxyl groups of L-malate, respectively, except that Thr105 always interacted with the carboxyl group away from the hydroxyl group (Figure 2B,C). The malate enantiomers in the two structures appeared in mirror symmetry. These observations indicate that both enantiomers bind at the same pocket, accompanied by subtle conformational changes.

**Figure 3:**
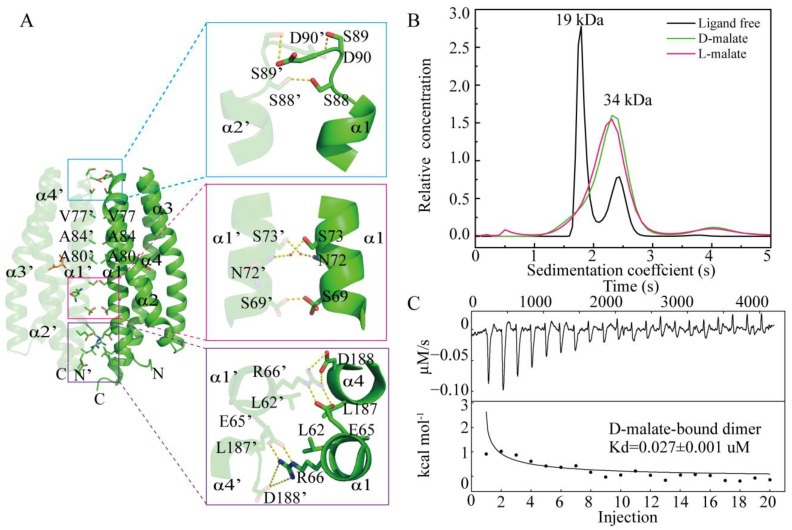
D-malate induces LBD dimerization as L-malate. (**A**) D-malate-bound dimeric interface. The two subunits are colored in light and dark colors. (**B**) Analytical ultracentrifugation assays of ligand-free, L-malate-bound, and D-malate-bound MCP2201 LBD. (**C**) ITC assays of MCP2201 LBD oligomer dissociation in the presence of D-malate. Calorimetric dilution data (top) for injection of MCP2201 LBD in the presence of 10 mM D-malate at 25 °C were integrated, and dilution-corrected peaks were fitted to an oligomer dissociation model (bottom) to assess the dissociation constants.

The structure indicated a D-malate-bound MCP2201 LBD dimer in the crystal. In the analytical ultracentrifugation assays, the D-malate-bound MCP2201 LBD predominately occurred as a dimer, the same as the L-malate-bound LBD (Figure 3B). The oligomer dissociation constant was determined as 0.027 ± 0.001 μM using isothermal titration calorimetry (ITC) assays (Figure 3C), which was comparable to that of the L-malate-bound dimer (Kd of 0.042 ± 0.005 μM). The dimeric interface of D-malate-bound MCP2201 LBD consists of α1 and α4 helices. Hydrophobic interactions are contributed by residues L62, V77, A80, A84, and L187 from both subunits, while hydrogen bonds are formed by residues E65, R66, S88, S89, D90, and D188 at the interface (Figure 3A). Additionally, residues S69, N72, and S73 participate in a water-mediated hydrogen bond network at the D-malate-bound dimeric interface. The dimeric interface was highly similar to that of the L-malate-bound dimer, with an r.m.s.d of 0.54 Å (Figure S2). Taken together, D-malate triggers a similar dimeric oligomerization to L-malate.

### 2.3. T105A Switches the Negative Chemotaxis Toward D-Malate to a Positive One

**Figure 4:**
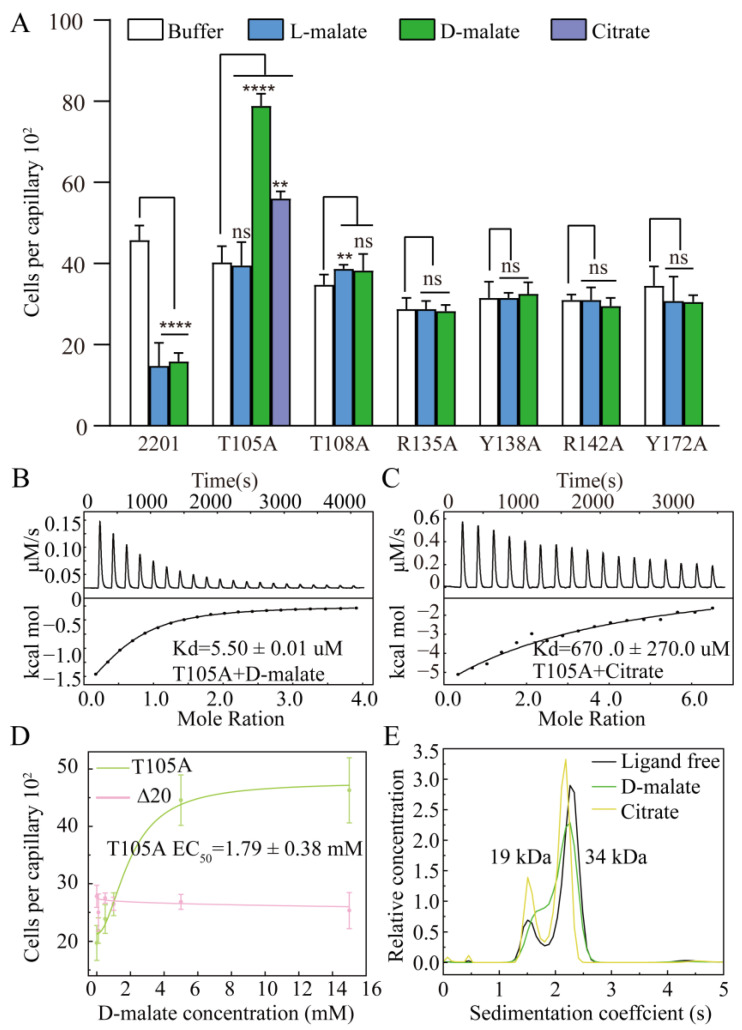
Chemotactic responses of MCP2201 mutants to D-malate. (**A**) Chemotactic responses of CNB-1Δ20 cells harboring MCP2201 mutants to L-malate, D-malate, and citrate in the capillary assay. Data are presented as mean ± SD (n = 6 biological replicates). Statistical significance was determined by an unpaired one-tailed Student’s *t*-test. ** (*p* < 0.01), and **** (*p* < 0.001) showed a significant difference between ligand-treated and non-treated groups. The “ns” stands for not significant. (**B**,**C**) D-malate (**B**) and citrate (**C**) affinity of T105A determined by isothermal titration calorimetry assays. (**D**) Chemotactic responses of Δ20 cells harboring T105A (green), or not (pink), toward different concentrations of D-malate in the capillary assay. Data were fitted into the logistic function. (**E**) Analytical ultracentrifugation assays of ligand-free, D-malate-bound, and citrate-bound T105A LBD. The experiments were repeated three times, and the representative instance is shown here.

We next performed site-directed mutations of those residues involved in ligand binding and assessed their chemotactic responses to D-malate using capillary assays. Mutants R135A, Y138A, R142A, and Y172A lost their chemotactic responses to D-malate, as well as those to L-malate, as previously reported (Figure 4A). Mutant T108A did not respond to D-malate and exhibited a weak chemotactic attraction ability to L-malate in both the previous and current studies [45]. Mutant T105A exhibited a chemoattractant response to D-malate, with an EC_50_ value of 1.79 ± 0.38 mM (Figure 4D). It did not respond to L-malate. These experiments demonstrate that the T105A mutation reverses MCP2201’s chemorepellent response to D-malate into a chemoattractant one and abolishes the ability to respond to L-malate.

A previous study indicated that the mutation of T105A (Kd is too weak to be determined for L-malate) and T108A (Kd of 995.1 ± 437.9 μM for L-malate) weakened the L-malate-binding affinities of LBD. Here, we conducted isothermal titration calorimetry (ITC) assays to verify the binding affinities of the T105A and T108A mutants for D-malate. The mutation of T108 into alanine abolished the ability to bind D-malate (Figure S1). In contrast, mutant T105A still bound D-malate with a Kd of 5.50 ± 0.01 μM, which suggested a higher D-malate affinity than that of the WT (40.0 ± 3.3 μM) (Figure 4B). These affinity assays agree that only T105A exhibited a strong response to D-malate.

We have reported that the chemoattractant (citrate) and chemorepellent (L-malate) induced the monomer–dimer equilibrium of MCP2201 LBD into a monomer (accompanied by a limited trimer) and a dimer, respectively, and triggered different swinging motions of signal helix α4-C [45]. The T105A mutation switched the chemotactic response to D-malate and abolished that toward L-malate, indicating a good case to study signal transduction. We checked T105A’s response to citrate using capillary assays and confirmed that it triggered a chemoattractant response to citrate, agreeing with the previously reported chemoattractant response in the gradient plate assays [45]. The citrate affinity of T105A was determined, with a dissociation constant (Kd) of 670.0 ± 270.0 uM (Figure 4C). Analytical ultracentrifugation assays were performed to check the oligomeric forms of T105A with or without ligand. Similarly to wildtype LBD, T105A occurred as the dynamic equilibrium of the monomer and dimer. In contrast, this also occurred as the equilibrium of monomer and dimer in the presence of D-malate or citrate, just like its ligand-free form (Figure 4E). This indicated that the mutation of T105 into alanine switched both the D-malate-bound dimer and the citrate-bound monomer (accompanied by a limited trimer) to the monomer–dimer equilibria.

### 2.4. The Variation in T105-Corresponding Residues Suggests the Diversity of Substrates Recognized by MCP2201 Similarities

**Figure 5:**
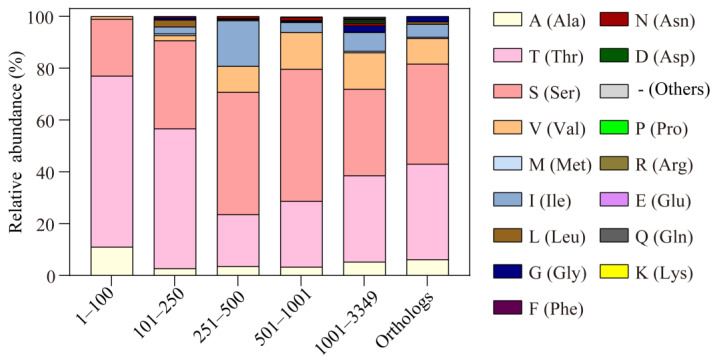
The relative abundances of alanine substitution of T105 in MCP2201 LBD similarities and different MCP2201 orthologs. The relative abundances of different substitutions of T105 in the top 1–100, 101–250, 251–500, 501–1001, and 1001–3349 sequences of LBD similarities and previously reported 201 MCP2201 orthologs are shown in a histogram.

We searched for the similarities of MCP2201 LBD in the non-redundant (nr) protein sequence database and checked the variation in T105-corresponding residues in different proteins. In the top 100 sequences with the lowest E values, 66% and 11% of those residues were threonine and alanine, respectively, and 22% of them were serine, a residue highly similar to threonine (Figure 5). Only 1% of them were valine. In the top 101–250, 251–500, 501–1000, and 1001–3349 sequences, 54.0%, 20.0%, 25.4%, and 33.3% of them were threonine, and 34.0%, 47.2%, 51.0%, and 33.4% were serine. The sums of threonine and serine were 88.0%, 57.2%, 76.4%, and 66.7%, respectively, suggesting that the majority of those protein sequences might recognize L- and D-malate, as well as citrate (Figure 5). Furthermore, 11.0%, 2.7%, 3.6%, 3.2%, and 5.3% of them were alanine, indicating that a small proportion of those proteins only recognize D-malate and citrate (Figure 5). In addition, the highest relative abundance of alanine found in the top 100 sequences reveals that the mutation of T105 into alanine in LBD mainly occurred in those sequences that are highly similar to MCP2201 LBD, indicating some manner of divergent evolution.

The abovementioned sequences were found based on the similarities in LBD and might not include the transmembrane helices, HAMP domains, and signaling domain, which were necessary for triggering chemotactic behavior. We, therefore, checked the T105 variation in the protein similarities of full MCP2201 reported in a previous study. In those 201 proteins, 37.1% and 38.6% of the T105-corresponding residues were threonine and serine, respectively, suggesting that most of the MCP2201 homologs would recognize L- and D-malate as chemorepellents and citrate as a chemoattractant. Furthermore, 5.9% of them were alanine, including the methyl-accepting chemotaxis protein from *Noviherbaspirillum massiliense* (accession number WP_019143412.1), *Cupriavidus* (WP_017511610.1), *Massilia* sp. Root335 (WP_056443095.1), *Collimonas fungivorans* (WP_061540704.1), *Acidovorax caeni* (WP_054257022.1), *Comamonas terrigena* (WP_066535939.1), *Duganella* sp. Root1480D1 (WP_082565276.1), *Duganella* sp. CF458 (WP_090439479.1), *Cupriavidus* sp. YR651 (WP_092142725.1), *Massilia alkalitolerans* (WP_084416306.1), and *Acidovorax* sp. 56 (WP_099657573.1). The sequence alignment shows that T104, T105, R135, Y138, and R142 and Y172 are highly conserved (Figure S4). It was suggested that these chemoreceptors might recognize D-malate and citrate as chemoattractants and do not respond to L-malate. These 12 proteins come from three families of Burkholderiales, i.e., Comamonadaceae, Burkholderiaceae, and Oxalobacteraceae (Figure S5). Similarly, these 201 sequences distribute across four orders of β- and γ-proteobacteria, and 95% of them distribute across four families of Burkholderiales (i.e., Comamonadaceae, Burkholderiaceae, Oxalobacteraceae, and Sphaerotilaceae) (Figure S6). The subtle distribution difference suggests that the substitution of T105 by alanine is not species-specific and appears random.

## 3. Discussion

Bacterial chemoreceptors, together with one-component systems, two-component systems, and so on, sense extracellular chemical and physical signals and help bacteria to respond and adapt to the changing environment. They can sense different chemical molecules, including amino acids [28], carbohydrates [29,30], tricarboxylic acids [31], phosphate [32], O_2_ [36,37,38], aromatic compounds [47], benzene [48], morphine [49], and so on [39,50]. The receptors Tar and Tsr from *E. coli* are the most extensively studied ones and used as models for studying bacterial chemotaxis; they have been shown to directly bind to L-aspartate and L-serine, respectively [51,52,53,54], without any other convincingly direct binding ligands. No evidence supports that they can bind to the enantiomer of L-aspartate and L-serine. Some bacterial chemoreceptors recognize a series of ligands, such as PctA (PA4309) from *P. aeruginosa* [41,42] and McpA from *P. putida*, which directly bind many amino acids [35,55], and TlpB (HP0103) from *H. pylori*, which directly binds urea, hydroxyurea, formamide, and acetamide [34]. Yet they have also not been reported to recognize the possible enantiomer of ligands. MCP2201 has been reported to bind the TCA intermediates, di- and tricarboxylic acids, and trigger positive and negative chemotactic responses to citrate and L-malate, respectively [44,45,46]. It mainly interacts with two carboxyl groups of ligands, illustrating why substrates must include at least two carboxyl groups. Here, we found that D-malate can bind to MCP2201 in the same pocket as L-malate binds. Mirror symmetry was observed for the bound L- and D-malate molecules, where the C1-C4 atoms of D-malate occupied the positions of the C4-C1 atoms of L-malate. These types of binding motifs ensure that the hydroxyl groups are in the same position, illustrating the reason why MCP2201 could bind both malate enantiomers.

In our previous study, we found that the ligand-binding pocket of MCP2201 underwent a significant conformational change upon ligand binding. For example, the atom Cα of T104 shifted away by 4.8 Å and 2.5 Å upon citrate and L-malate binding, respectively, whereas those of T108 shifted by 2.9 Å and 1.4 Å [45,46]. The D-malate-bound structure is almost identical to the L-malate-bound one and causes the same conformational change compared to the apo form. In Tar, the ligand-binding pockets were almost identical to each other in the ligand-free and -bound forms [56]. In PctA, the ligand-binding pocket did not undergo a significant conformational change upon binding different ligands [57]. The much more significant local conformational change upon ligand binding in MCP2201 compared to that in Tar and PctA demonstrated the flexibility of the binding pocket in MCP2201. The recognition of both malate enantiomers further suggested that the binding pocket of MCP2201 is flexible enough to accommodate many more ligands. Such a flexible ligand-binding pocket is an excellent template for evolving new substrate specificity via mutagenesis, which should be helpful for biosensor design.

The finding of D-malate as the repellent for MCP2201 actually provides an opportunity to examine the signal transduction mechanism proposed in a study of repellent L-malate [45]. The C-terminus of helix α4, however, was involved in the crystal packing (Figure S3), which made it difficult to assess the role of its orientation in signal transduction. Instead, D-malate binds to the same pocket and induces the same dimerization of LBD as L-malate, suggesting the same signal generation and transduction for D- and L-malate-bound LBD. We hypothesize that the dimerization of LBD would increase the orderliness of the chemotaxis array, which could be essential for enhancing the kinase activity of CheA and, subsequently, for the regulation of motility to generate a repellent response.

We noticed that the substitution of T105 with alanine abolished the negative chemotaxis toward L-malate, weakened the positive chemotaxis toward citrate, and switched the negative chemotaxis toward D-malate to a positive response. This mutation did not alter the monomer–dimer equilibrium of ligand-free LBD yet switched both the citrate-bound monomer (with a limited trimer) and the D-malate-bound dimer to a monomer–dimer equilibrium. In other words, the T105A mutation abolished the regulatory ability of LBD oligomeric organizations and retained the response ability for some chemical molecules, though there was a switch from a negative to a positive response. Considering that both MCP2201 and the T105A mutant mediated the chemoattractant responses toward citrate and that LBD and the T105A mutant existed as a monomer and a monomer–dimer equilibrium, respectively, we thus propose that the oligomerization of LBD is a consequence of signal generation rather than a prerequisite for transmembrane signaling. This hypothesis agrees with the observations that different LBDs in different bacteria exist as different oligomeric states and undergo different oligomerization switches, or not, upon ligand binding [43,58,59]. The observation that a single mutation, T105A, causes a change in the substrate range and response type also provides a chance to explore protein evolution. We found that some MCP2201 homologs, as well as some LBD homologs, carry an alanine residue at the equivalent site of T105. In most of these homologs, threonine or a structurally similar residue, such as serine, is typically present at this site. This finding suggests both MCP2201 similarities and LBD homologs have already undergone differentiation in substrate ranges and protein functions. The alanine substitution mainly occurs in those sequences highly similar to MCP2201, a possible consequence of divergent evolution.

## 4. Materials and Methods

### 4.1. Strains, Plasmids, Media, and Cultivation

All bacterial strains and plasmids used in this study are listed in Supplementary Table S1. Genetic complementation in *Comamonas testosteroni* CNB-1 [60] was conducted using pBBR1MCS2 [61]. *Comamonas testosteroni* CNB-1 and mutants were cultivated in Luria–Bertani (LB) broth or on LB plates with 1.5% (*w*/*v*) agar (Lablead, Beijing, China) at 30 °C.

### 4.2. Construction of the Mutants

The plasmid pBBR1MCS2 harboring MCP2201 (pBBR1MCS2-*mcp2201*) was amplified with designed point mutation primers and digested with DpnI (NEB, Ipswich, MA, USA) for 2.5 h. Then, 4 µL of the digested solution was transformed into *E. coli* DH5α cells. The correct point mutant was confirmed by DNA sequencing.

### 4.3. Gradient Soft-Agar Swim Plate Assay

The semisolid agar assay was performed following previously reported protocols [62,63]. Briefly, 1.5% (*w*/*v*) agar plugs containing 10 mM D-malate (Sigma-Aldrich, St. Louis, MO, USA) were placed at the center of a 0.25% (*w*/*v*) semisolid agar Petri dishes with 1 mM citrate (Sigma-Aldrich, St. Louis, MO, USA) in Motility Semi-solid Broth (MSB medium) (1 g L^−1^ Na_2_HPO_4_·12H_2_O, 0.5 g L^−1^ KH_2_PO_4_, 0.03 g L^−1^ MgSO_4_·7H_2_O, and 1 g L^−1^ NH_4_Cl, pH 8.0). When the optical density at 600 nm (OD_600_) reached 0.8, 1 mL of *C. testosteroni* CNB-1 culture was harvested, washed, resuspended in 50 µL of MSB medium, and inoculated 2 cm away from the center of the agar plugs. After incubation at 30 °C for 24 h, the distances from the inoculation sites to the edges of the colony closest (D1) to and furthest (D2) from the center of the agar plug were measured and used to calculate the response index (RI) values using RI = D1/(D1 + D2), as described previously [60]. RI values greater than 0.52 and less than 0.48 indicate the attractant and repellent responses, respectively.

### 4.4. Capillary Assay

A capillary assay was performed following the previously reported protocol [64]. Overnight cultures of bacteria were grown in LB medium, after which, 1% of the culture was transferred to fresh LB medium and incubated at 30 °C with shaking at 180 rpm until the OD_600_ reached 0.7–1.0. The cultures were then harvested by centrifugation at 5000× *g*, washed three times with MSB medium, and resuspended in MSB medium to a final OD_600_ of 0.8–1.0. The bacterial suspension was placed in a 15 mL tube (ACMEC, Shanghai, China) to ensure consistent OD values in each well of a multi-channel pipette. Then, 100 µL of D-malate was added to a 1 mL syringe (STEEMA, Beijing, China), and the syringe needle was inserted into one end of the pipette. After incubation at room temperature for 1 h, the pipette containing the bacterial suspension was removed, and the contents of the syringe were injected into 900 µL of MSB medium. The collected samples were streaked and incubated overnight for colony-forming unit (CFU) counting. Each treatment was repeated ten times.

### 4.5. Protein Expression and Purification

The coding sequence for MCP2201 LBD (residues 58–203) was cloned in pET-28a expression vector (Merck, Darmstadt, Germany) with an N-terminal His6-tag. The recombinant plasmid was transformed into *E. coli* BL21 (DE3) (Vazyme, Beijing, China). The cells were cultured in LB medium containing 50 µg mL^−1^ kanamycin (Coolaber, Beijing, China) at 37 °C. When the OD_600_ reached 1.0, protein expression was induced with 0.3 mM IPTG, followed by incubation at 16 °C for 18 h. The cells were harvested by centrifugation at 5000× *g*, resuspended in lysis buffer (20 mM Tris buffer (pH 7.5), 200 mM NaCl, and 10 mM imidazole), lysed on ice by sonication (SCIENTZ, Ningbo, China), and centrifuged at 15,000× *g*. The supernatant was loaded on nickel affinity resins (Ni-NTA, Qiagen, Dusseldorf, Germany) for five repeated cycles. The resin was washed five times with washing buffer containing 20 mM Tris (pH 7.5), 300 mM NaCl, and 30 mM imidazole. The protein was eluted using elution buffer containing 20 mM Tris (pH 7.5), 200 mM NaCl, and 300 mM imidazole. The eluate was further purified using a Superdex 75 10/300 GL size-exclusion chromatography column (GE Healthcare, Pittsburgh, PA, USA) and eluted with buffer containing 20 mM Tris (pH 7.5) and 150 mM NaCl. Fractions containing the target protein were concentrated to 8–10 mg mL^−1^ for crystallization screening and additional assays.

### 4.6. Crystallization, Data Collection, and Structure Determination

D-malate-bound MCP2201 LBD crystals were obtained in a solution containing 1.8 M ammonium sulfate and 5% glycerol using the hanging-drop vapor diffusion method at 289 K. X-ray diffraction data were collected at the BL19U1 beamlines of the Shanghai Synchrotron Radiation Facility (SSRF, Shanghai, China). iMOSFLM (Versuion 7.2.2, Heidelberg, Germany) [65], XDS (Versuion 19 September 2022, Heidelberg, Germany) [66], and/or CCP4 suite (Versuion 0.9.8.1, Warrington, UK) [67] were used to process and scale the data. The structure was solved by molecular replacement using Phaser in the PHENIX suite (Versuion 1.14-3260, Berkeley, CA, USA) [68], with the ligand-free MCP2201 LBD structure (PDB entry 5XUA) as the search model. Structure refinement was carried out using PHENIX (Versuion 1.14-3260, Berkeley, CA, USA) [68]. Data collection and refinement statistics are listed in Table S2. Figures were generated using PyMOL (http://pymol.org, accessed on 10 September 2024).

### 4.7. Isothermal Titration Calorimetry

ITC experiments were conducted using an Affinity ITC (TA Instruments, New Castle, DE, USA) at 25 °C. To determine the dimer dissociation constant, 1.1 mM protein in buffer containing 10 mM D-malate, 20 mM Tris (pH 7.5), and 150 mM NaCl was injected into the sample cell containing the same buffer. To assess ligand binding affinity, 100 μM of protein in the sample cell was titrated with 1.5 mM of D-malate dissolved in 20 mM of Tris (pH 7.5) and 150 mM of NaCl. Data were analyzed by the NanoAnalyze software (Versuion 3.11.0) package (TA Instruments, New Castle, DE, USA).

### 4.8. Analytical Ultracentrifugation Assay

The sedimentation coefficients and apparent molecular masses of MCP2201 LBD and T105A LBD were determined using a Beckman XL-I analytical ultracentrifuge (Beckman Coulter, Brea, CA, USA) operated at 60,000× *g* for 7 h at 4 °C. Data analysis was performed as previously described [69].

### 4.9. Protein Sequence Alignment

Protein sequence alignment was performed using MEGA (Molecular Evolutionary Analysis, Versuion 11.0.13, Mega Limited, Auckland, New Zealand) with ClusterW (https://www.genome.jp/tools-bin/clustalw, accessed on 19 September 2024) [70]. Figures were prepared using ESPript 3.0 (https://espript.ibcp.fr/ESPript/ESPript/index.php, accessed on 19 September 2024) [71].

## 5. Conclusions

Our study identifies D-malate as a novel chemorepellent for the bacterial chemoreceptor MCP2201, exhibiting functional and structural parallels to L-malate. Experimental assays confirmed that D-malate elicits a repellent chemotactic response. Crystallographic analysis revealed that D- and L-malate occupy the same binding pocket in a mirror-symmetric fashion, engaging conserved residues and inducing LBD dimerization through a shared structural mechanism. Notably, a single point mutation (T105A) reversed the chemorepellent response to D-malate into attraction, enhanced binding affinity, and disrupted ligand-dependent oligomerization while abolishing the response to L-malate. Evolutionary analysis showed that this residue is conserved as threonine or serine in most homologs, supporting dual ligand recognition, whereas alanine variants—naturally found across diverse Burkholderiales lineages—favor D-malate and citrate specificity, suggesting a case of convergent functional evolution. Moreover, the discovery that such mutations are naturally distributed across taxonomically diverse bacteria points to a broader evolutionary strategy for tuning ligand specificity and signaling mode.

## Data Availability

The structure factor and coordinate files have been deposited in the Protein Data Bank under the accession code number 9KUP. The other data generated or analyzed during this study are included in this published article and its Supplementary Information files.

## Supplementary Materials

The following supporting information can be downloaded at https://www.mdpi.com/article/10.3390/ijms26104902/s1.
