# Supplementary material for: Insights into Chemoreceptor MCP2201-Sensing D-Malate
Source: Int J Mol Sci. 2025 May 20;26(10):4902. doi: 10.3390/ijms26104902 (PMC12112095; doi:10.3390/ijms26104902)
Supplement: Supplementary file 1 [file ijms-26-04902-s001.zip › ijms-3638232-supplementary.pdf]

Supplementary Information

Supplement to: "Insights into chemoreceptor MCP2201 sensing D-malate"

Rui Cui [1,2], Jie Li [1,2], Yuan Hong [3], Lu Guo [1], Yun-Hao Wang [4], Yi-Fei Bai [1] and De-Feng Li [1,2,*]

Contents

Figures S1 to S6

Tables S1 to S2

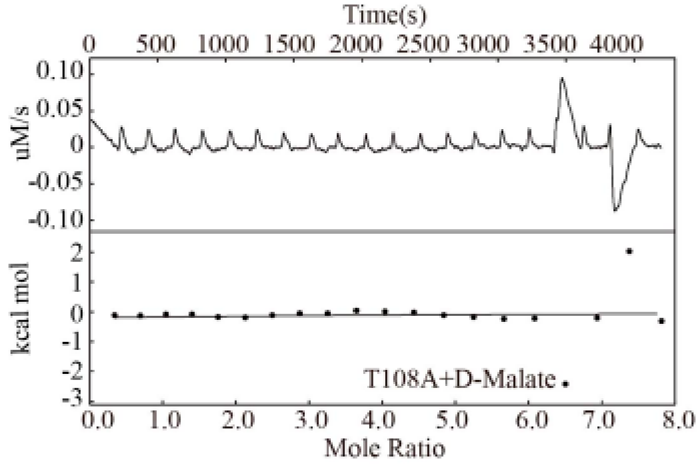

Figure S1. The D-malate affinity of T108A measured by isothermal titration calorimetry assays.

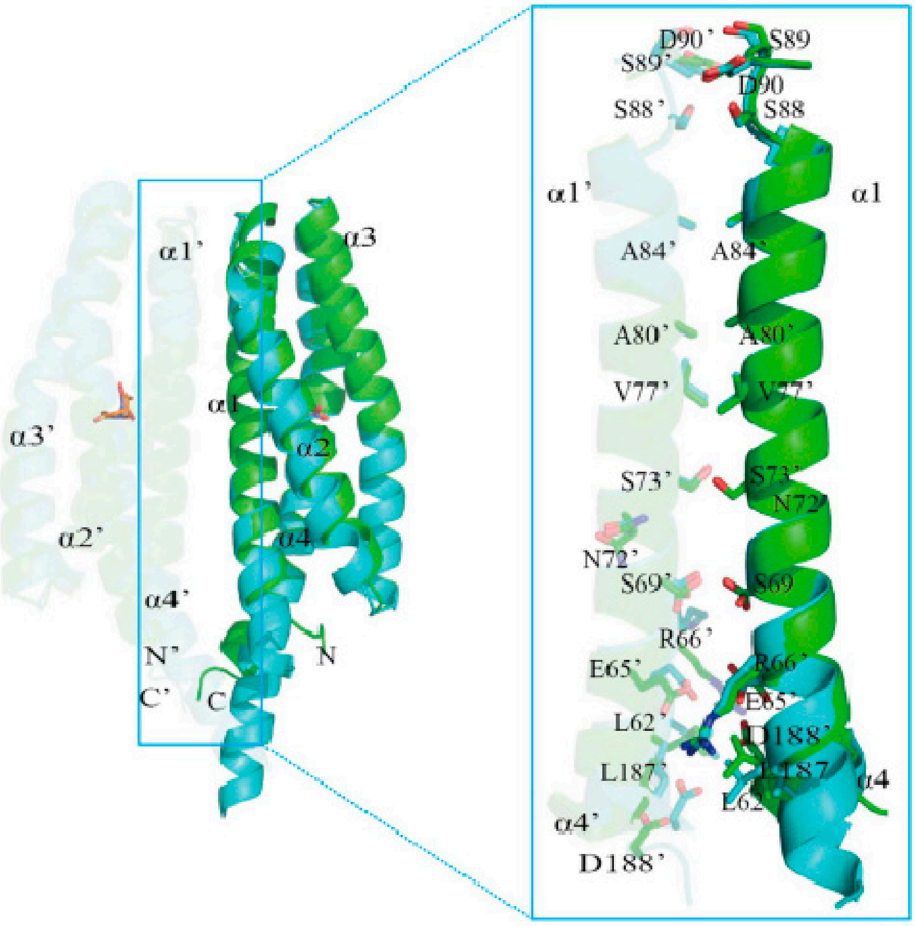

Figure S2. Structural superposition of the D- and L-malate-bound dimers and those residues involved in the dimeric interface. D-malate (green)) and L-malate-bound (blue) LBD were superposed and shown in cartoon. Two subunits were colored in light and dark.

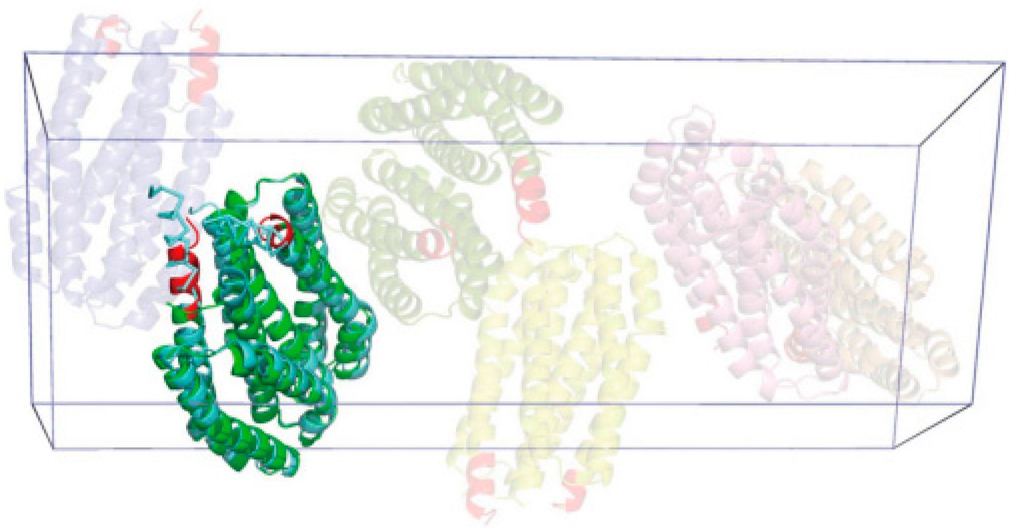

Figure S3. The C-terminus of helix α4 is involved in the crystal packing. The unit cell is shown in purple box. Each asymmetric unit is shown in different color. A L-malate-bound dimer (cyan) is superposed with one asymmetric unit. The C-terminus of helix α4 is in red.

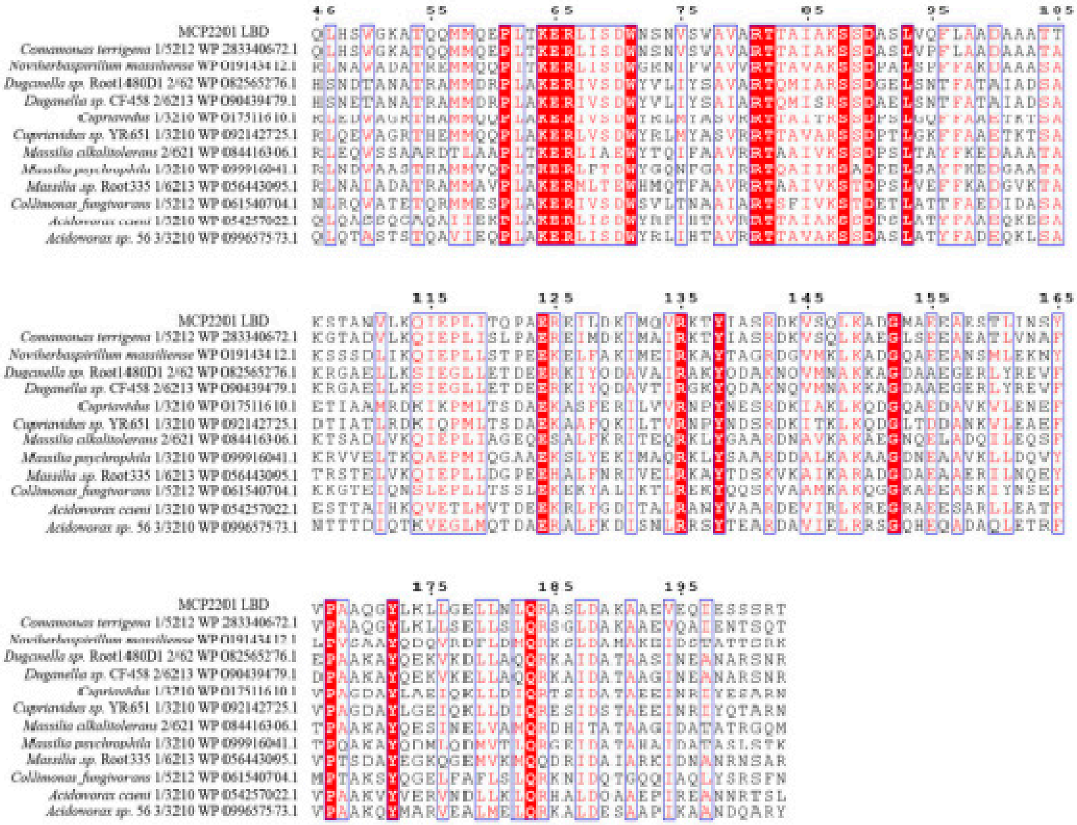

Figure S4. Multiple sequence alignment of MCP2201 similarities harboring the alanine substitution of T105. The identical residues are highlighted with red.

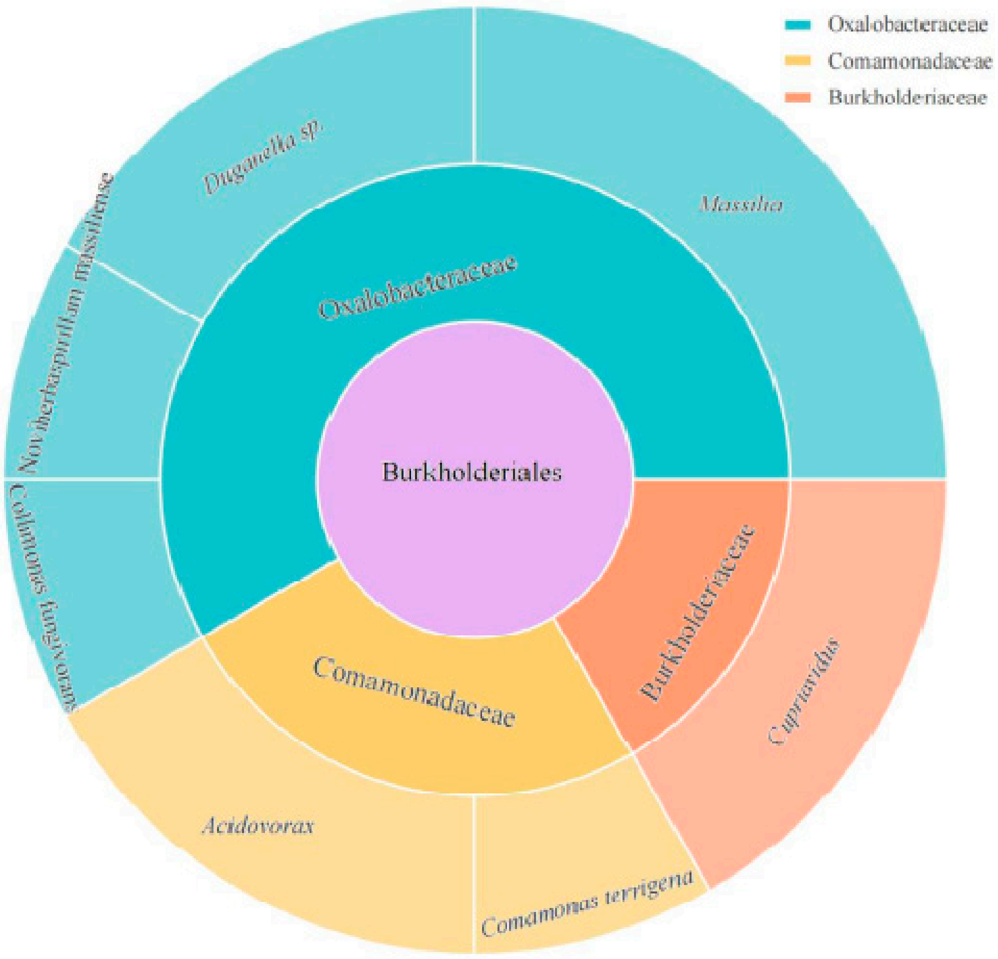

Figure S5. The order, family and genus distribution of those MCP2201 similarities harboring the T105A substitution of T105. The outer, mediate, and inner circles represent the relative of genus, family, and order, respectively.

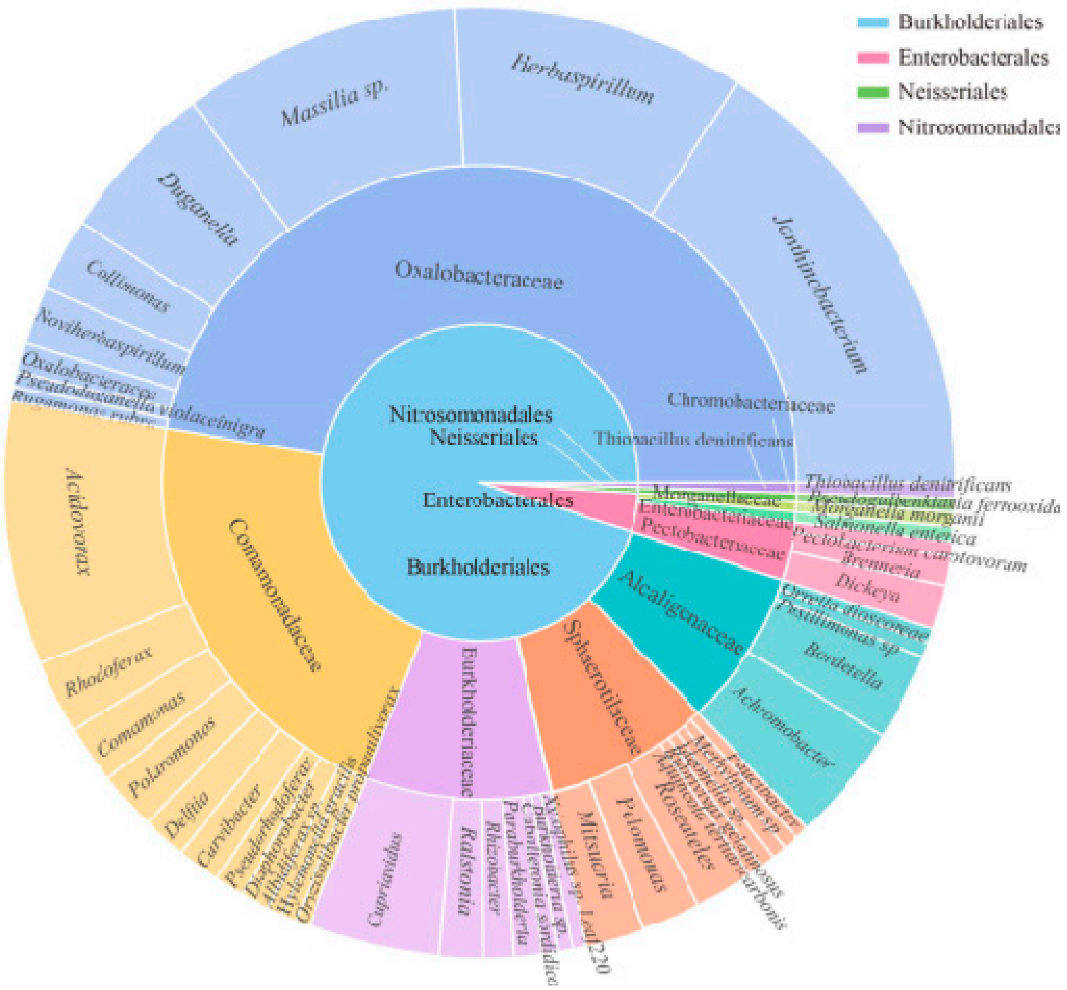

Figure S6. The order, family and genus distribution of those previously reported 201 MCP2201 orthologous. The outer, mediate, and inner circles represent the relative of genus, family, and order, respectively.

Table S1. Bacterial strains and plasmids used in this study.

Strains/plasmids	Description	Source
**Strains**		
Comamonas testosteroni		
CNB-1		[1]
CNB-1Δ20	All putative chemoreceptor genes were disrupted in strain CNB-1	[2]
Escherichia coli		
DH5α	F^- *φ*80d *lacZ*ΔM15 Δ (*lacZYA-argF*) U169 *rec*A1 *end*A1 *hsd*R17(r_K^- m_K^+) *sup*E44 λ- *thi*-1 *gyr*A96 *rel*A1 *pho*A; host for DNA manipulations	[3]
BL21(DE3)	F^- *omp*T *hsd*S_B(r_B^- m_B^-) *gal dcm* (DE3)	Vazyme,China
**Plasmids**		
pBBR1MCS2	Km^r, *lacPOZ'* broad host vector with R type conjugative origin	[4]
pBBR1MCS2-MCP2201-T105A	Carries MCP2201 with E65AR66A mutation	This work
pBBR1MCS2-MCP2201-R81A	Carries MCP2201 with S69A mutation	This work
pBBR1MCS2-MCP2201-T104A	Carries MCP2201 with N72A mutation	This work
pBBR1MCS2-MCP2201-T108A	Carries MCP2201 with S73A mutation	This work
pBBR1MCS2-MCP2201-R135A	Carries MCP2201 with S73F mutation	This work
pBBR1MCS2-MCP2201-Y142A	Carries MCP2201 with S73R mutation	This work
pBBR1MCS2-MCP2201-R138A	Carries MCP2201 with V77W mutation	This work
pBBR1MCS2-MCP2201-Y172A	Carries MCP2201 with A80F mutation	This work
pBBR1MCS2-MCP2201-A80W	Carries MCP2201 with A80W mutation	This work
pBBR1MCS2-MCP2201-A84F	Carries MCP2201 with A84F mutation	This work
pBBR1MCS2-MCP2201-A84W	Carries MCP2201 with A84W mutation	This work
pET28a		
pET28a-*mcp2201* LBD	pET28a derivative for expression of MCP2201 LBD, spanning residues 58-203 and used for D-malate-bound form structure determination.	This work
pET28a-*mcp2201* LBD-T105A	pET28a derivative for expression of MCP2201 LBD-T105A	This work
pET28a-*mcp2201* LBD-T108A	pET28a derivative for expression of MCP2201 LBD-T108A	This work

Table S2. Data collection and refinement statistics

	MCP2201 LBD (D-malate-bound)
**Data collection**	
Space group	P 3_1 2 1
Cell dimensions	
a, b, c (Å)	59.80, 59.80, 158.20
α, β, γ (°)	90, 90, 90
Resolution (Å)	43.33 – 1.50 (1.55 – 1.50)
R_{merge}	0.11 (0.39)
I/σ(I)	18.6 (4.4)
$CC_{1/2}$	99.7 (92.4)
Completeness (%)	94.7 (72.2)
Redundancy	17.5 (8.0)
**Refinement**	
No. reflections	26185
R_{work}/R_{free}	0.16 / 0.19
No. Non-H atoms	
Protein	2476
Ligand/ion	34
Water	397
B factors	
Protein	18.34
Ligand/ion	17.91
Water	34.53
R.m.s. deviations	
Bond lengths (Å)	0.01
Bond angles (°)	1.28
Ramachandran plot	
Favored	98.86%
Allowed	1.14%
Outliers	0.00%

Values in parentheses refer to the highest resolution shell.

References

1. Wu, J.; Jiang, C.; Wang, B.; Ma, Y.; Liu, Z.; Liu, S. Novel partial reductive pathway for 4-chloronitrobenzene and nitrobenzene degradation in *Comamonas sp.* strain CNB-1. *Appl Environ Microbiol* **2006**, *72*, 1759–1765, doi:10.1128/AEM.72.3.1759-1765.2006.
2. Ni, B.; Huang, Z.; Fan, Z.; Jiang, C.-Y.; Liu, S.-J. *Comamonas testosteroni* uses a chemoreceptor for tricarboxylic acid cycle intermediates to trigger chemotactic responses towards aromatic compounds. Mol Microbiol 2013, 90, 813–823, doi:10.1111/mmi.12400.
3. Hanahan, D. Studies on transformation of *Escherichia Coli* with plasmids. *J Mol Biol* **1983**, *166*, 557–580, doi:10.1016/s0022-2836(83)80284-8.
4. Kovach, M.E.; Elzer, P.H.; Hill, D.S.; Robertson, G.T.; Farris, M.A.; Roop, R.M.; Peterson, K.M. Four new derivatives of the broad-host-range cloning vector pBBR1MCS, carrying different antibiotic-resistance cassettes. *Gene* **1995**, *166*, 175–176, doi:10.1016/0378-1119(95)00584-1.
